# Role of ClC-K and barttin in low potassium-induced sodium chloride cotransporter activation and hypertension in mouse kidney

**DOI:** 10.1042/BSR20171243

**Published:** 2018-01-30

**Authors:** Naohiro Nomura, Wakana Shoda, Yuanlong Wang, Shintaro Mandai, Taisuke Furusho, Daiei Takahashi, Moko Zeniya, Eisei Sohara, Tatemitsu Rai, Shinichi Uchida

**Affiliations:** Department of Nephrology, Graduate School of Medical and Dental Sciences, Tokyo Medical and Dental University, 1-5-45 Yushima, Bunkyo, Tokyo 113-8519, Japan

**Keywords:** chloride, hypertension, intracellular signaling, molecular basis of health and disease, renal physiology, transport

## Abstract

The sodium chloride cotransporter (NCC) has been identified as a key molecule regulating potassium balance. The mechanisms of NCC regulation during low extracellular potassium concentrations have been studied *in vitro.* These studies have shown that hyperpolarization increased chloride efflux, leading to the activation of chloride-sensitive with-no-lysine kinase (WNK) kinases and their downstream molecules, including STE20/SPS1-related proline/alanine-rich kinase (SPAK) and NCC. However, this mechanism was not studied *in vivo*. Previously, we developed the barttin hypomorphic mouse (*Bsnd^neo/neo^* mice), expressing very low levels of barttin and ClC-K channels, because barttin is an essential β-subunit of ClC-K. In contrast with *Bsnd^−/−^* mice, *Bsnd^neo/neo^* mice survived to adulthood. In *Bsnd^neo/neo^* mice, SPAK and NCC activation after consuming a low-potassium diet was clearly impaired compared with that in wild-type (WT) mice. In *ex vivo* kidney slice experiment, the increase in pNCC and SPAK in low-potassium medium was also impaired in *Bsnd^neo/neo^* mice. Furthermore, increased blood pressure was observed in WT mice fed a high-salt and low-potassium diet, which was not evident in *Bsnd^neo/neo^* mice. Thus, our study provides *in vivo* evidence that, in response to a low-potassium diet, ClC-K and barttin play important roles in the activation of the WNK4-SPAK-NCC cascade and blood pressure regulation.

## Introduction

Hypertension is a major worldwide public health problem associated with a variety of complications including stroke, heart failure, and kidney failure. Diets play a strong contributory role in blood pressure. Dietary potassium (K^+^) intake is highly related to blood pressure and mortality, and recent studies have shown that K^+^ intake was inversely related to blood pressure [1–3]. The sodium (Na^+^) chloride (Cl^−^) cotransporter (NCC) expressed in the distal convoluted tubules (DCT) in the kidney plays an important role in the regulation of urinary K^+^ excretion, as well as in blood pressure regulation by NaCl reabsorption. In previous animal studies, a low-K^+^ diet increased the total amount and the phosphorylation of NCC [4–9] and elevated blood pressure [4,10]. This elevation of blood pressure with a low-K^+^ diet was dependent upon NCC, because NCC^−/−^ mice did not show an elevation of blood pressure when under a low-K^+^ diet [4]. Although NCC itself does not directly transport K^+^, the amount of NaCl reabsorption in the DCT affects the delivery of Na^+^ to the downstream cortical collecting ducts where K^+^ is excreted, based upon Na^+^ reabsorption via epithelial Na^+^ channels. The notion that the NCC is important for regulating K^+^ excretion is also supported by the facts that two genetic diseases, Gitelman syndrome (caused by the loss-of-function of NCC) and pseudohypoaldosteronism type II (caused by the gain-of-function of NCC) show hypokalemia and hyperkalemia, respectively [11,12].

With-no-lysine kinase (WNK) kinases phosphorylate the STE20/SPS1-44 related proline-alanine-rich protein kinase (SPAK) and the related oxidative stress-related kinase 1 (OSR1), which directly activate NCC [13,14]. It has been proposed that the phosphorylation of NCC with a low-K^+^ diet is dependent upon WNK4 and OSR1/SPAK kinases. Low K^+^ activates not only NCC but also WNKs and SPAK [4,7,8,10]. Furthermore, WNK4^−/−^ and SPAK^−/−^ mice showed either no increase or only a mild increase, in pNCC in response to a low-K^+^ diet, respectively [4,7–9,15]. Disrupting both SPAK and OSR1 almost completely ablated the response of pNCC to dietary K^+^ restriction [4,9]. The mechanism by which low K^+^ activates WNKs was previously investigated in cultured cells and postulated as follows: a decrease in extracellular K^+^ concentration ([K^+^]_ex_) affects the membrane potential of cells, thereby decreasing intracellular Cl^−^ concentration ([Cl^−^]_i_) with Cl^−^ efflux via a Cl^−^ channel [4]. Since WNK kinases are regulated by [Cl^−^]_i_ [15,16], this decrease in [Cl^−^]_i_ by low [K^+^]_ex_ activates WNK signaling. As for the molecular identity of the channel responsible for the Cl^−^ efflux in DCT, ClC-Kb (a human homolog of ClC-K2) chloride channel has been postulated due to its localization in the DCT [17,18]. Additional genetic evidence showed that Barter syndrome type III caused by the loss-of-function mutations in the *CLCKB* gene (which codes ClC-Kb) had similar phenotypes to Gitelman syndrome [19,20]. Further, a recent study using patch clamp analysis of tubules isolated from the ClC-K2 knockout mouse reported that ClC-K2 is the predominant Cl^−^ channel on the basolateral membrane of DCT cells [21]. Indeed, Terker et al. [4] prepared HEK293 cells overexpressing wild-type (WT) or loss-of-function mutant ClC-Kb, and they found that low-K^+^-induced phosphorylation of NCC was attenuated by the mutation suggesting the potential role of ClC-Kb in regulating [Cl^−^]_i_ in the DCT cells. However, these data were obtained only from *in vitro* cultured cell studies. To clarify the contribution of ClC-K2 in the mechanism of NCC phosphorylation in response to K^+^ restriction *in vivo*, we performed animal studies. Previously, we generated *Bsnd^neo(R8L)/neo(R8L)^* (*Bsnd^neo/neo^*) mice, which are hypomorphic of a disease-causing mutant barttin (R8L barttin) [22]. Barttin (coded by the *Bsnd* gene) is an essential β-subunit for both ClC-Ka/1 and ClC-Kb/2 channels [23,24]. Since barttin is crucial for ClC-K membrane localization and stability, the genetic ablation of barttin resulted in a ClC-K knockout condition [25]. We used *Bsnd^neo/neo^* mice to investigate the role of ClC-K and barttin in NCC activation by low-K^+^ diet.

## Materials and methods

### Animal experiments

All experiments were performed in accordance with the guidelines for animal research of Tokyo Medical and Dental University, and the protocol was approved by The Animal Care and Use Committee of Tokyo Medical and Dental University. Studies were performed on *Bsnd^neo/neo^* mice (C57BL6 background) as a loss-of-function model for ClC-K [22]. Littermates WT mice or C57BL/6 mice (Japan SLC, Inc., Hamamatsu, Japan) were used for WT control mice. We used mixed sex, 20–30 g body weight, 10–16 weeks old mice. These parameters were matched in each group. A high-salt and normal-K^+^ diet (6% NaCl, 1% K^+^, a high-salt and normal-K (HSNK) diet) and a high-salt and low-K^+^ diet (6% NaCl, 0.01% K^+^, a high-salt and low-K (HSLK) diet) were prepared by adding NaCl and KCl (or sucrose for adjustment of volume) to a K^+^-deficient diet which was modified from AIN-76 diet (Oriental Yeast Co., Tokyo, Japan). Blood was collected from the retro-orbital venous plexus under anesthesia. Blood data were analyzed by iSTAT EC8+ (Abbott, Inc., Abbott Park, IL). Serum aldosterone levels were measured by the SRL clinical laboratory service (Tokyo, Japan). Noninvasive systolic blood pressures were measured by a programmable tail-cuff sphygmomanometer (MK-2000A, Muromachi Kikai Co., Tokyo, Japan) with the investigator blinded for the treatment groups. Systolic blood pressure of mice consuming an HSNK diet was measured after a 2-day period of acclimation to the instrument. Blood pressure was recorded again 2 days after switching diets from an HSNK diet to an HSLK diet. The changes in blood pressure after the switching diets were compared between WT mice and *Bsnd^neo/neo^* mice with unpaired *t* test. Each blood pressure data point was calculated as the mean of ~20 sequential measurements.

### Western blotting and immunofluorescence

Immunoblotting and immunofluorescence were performed as previously described [7]. The detail of the method is described in Supplementary information. Briefly, kidneys were homogenized and then the homogenates were centrifuged to separate entire kidney samples without the nuclear fraction, as either whole kidney lysates (600 g, supernatant) and crude membrane fraction (17000 g, pellet). The membrane fractions were used for the analysis of barttin, ClC-K, pNCC and total NCC (tNCC). The whole kidney lysates were used for the WNK4, pSPAK and tSPAK detection. The relative intensities of immunoblot bands were analyzed and quantitated using ImageJ software (National Institutes of Health, Bethesda, MD). For the experiment of correlation between pNCC and plasma K^+^ concentration, each value of pNCC/tNCC from HSLK groups were compared with their internal control group on an HSNK diet. pNCC/tNCC average of the internal control group was set to 1.

For immunofluorescence, kidneys were fixed by perfusion through the left ventricle with periodate lysine (0.2 M) and paraformaldehyde (2%) in PBS. Tissue samples were soaked for several hours in 20% sucrose in PBS, embedded in Tissue Tek O.C.T. Compound (Sakura Finetechnical Co., Ltd, Tokyo, Japan), and frozen in liquid nitrogen.

Antibodies are listed in Supplementary Table S1. The primary antibodies used in the present study were as follows: rabbit anti-WNK4 [7], rabbit anti-pSPAK (Ser^383^, kindly gifted by Dr S.S. Yang, National Defense Medical Center) [26], rabbit anti-total SPAK (Cell Signaling Technology, Inc., Danvers, MA, #2281), rabbit anti-pNCC (Ser^71^) [27], rabbit anti-tNCC [7], guinea pig anti-tNCC [7,22], rabbit anti-ClC-K (kindly gifted by Dr T.J. Jentsch, Max-Delbrck-Centrüm für Molekulare Medizin) [25], and rabbit anti-actin antibody (Cytoskeleton, Inc. Denver, CO. AAN01, Lot 121). We raised a new anti-pSPAK antibody in rabbit against the synthetic peptide, which recognizes the same phosphorylation site as the previous one [26]. The band of WNK4, pSPAK and total SPAK had been confirmed by using the knockout mice (Supplementary Figure S1) [7]. We used two different pSPAK antibodies because we had used up the previous one. As for secondary antibodies, alkaline phosphatase-conjugated anti-IgG antibodies (Promega Corporation, Fitchburg, WI) and Alexa 488 or 546 dye-labeled antibodies (Molecular Probes, Inc., Eugene, OR) were used for Western blotting and immunofluorescence, respectively. Western Blue® (Promega Corporation, Fitchburg, WI) was used to detect the signals and immunofluorescence images were acquired using the LSM510 Meta confocal microscope (Carl Zeiss, Oberkochen, Germany). The linearity of protein detection for each antibody was confirmed in Supplementary Figures S2 and S3.

### *Ex vivo* kidney slice experiment

Kidney slices were prepared as described previously [28]. Kidney slices of less than 0.5 mm were cut using a microslicer (Natume Seisakusho Co., Ltd, Tokyo, Japan) on ice-cold Hank’s buffer medium (110 mM NaCl, 3 mM KCl, 1.2 mM MgSO_4_, 1.8 mM CaCl_2_, 4 mM Na acetate, 1 mM Na citrate, 6 mM d-glucose, 6 mM l-alanine, 1 mM NaH_2_PO_4_, 3 mM Na_2_HPO_4_, 25 mM NaHCO_3_). All the sliced kidneys were incubated in the Hank’s buffer medium at room temperature for 20 min. After the recovery in the Hank’s buffer medium for 20 min, the slices from the same kidney were separated in normal-K^+^ (4 mEq/l) or low-K^+^ (2 mEq/l) medium, then incubated for 30 min at 28°C. Different K^+^ concentration medium was prepared by using KCl or choline chloride to maintain the same Cl^−^ concentration in each medium. During the experiments, all solutions were continuously bubbled with 95% O_2_ and 5% CO_2_. After the incubation, slices were snap-frozen in liquid nitrogen and processed for immunoblotting as same as for whole kidney samples described above and Supplementary information. After Western blotting, the fold change of the band density in a low-K^+^ medium to the one in a normal medium were analyzed.

### Statistical analysis

Data were presented as the means ± S.E.M. Two-way ANOVA and Bonferroni’s post test was used to compare the multiple groups while the *t*test was used to compare two groups. For all analyses, a *P*-value <0.05 was considered to be statistically significant. For correlation analyzes, Pearson’s test was performed.

## Results

### *Bsnd^neo/neo^* mice showed very low amount of barttin and ClC-K and no change in NCC phosphorylation

**Figure 1:**
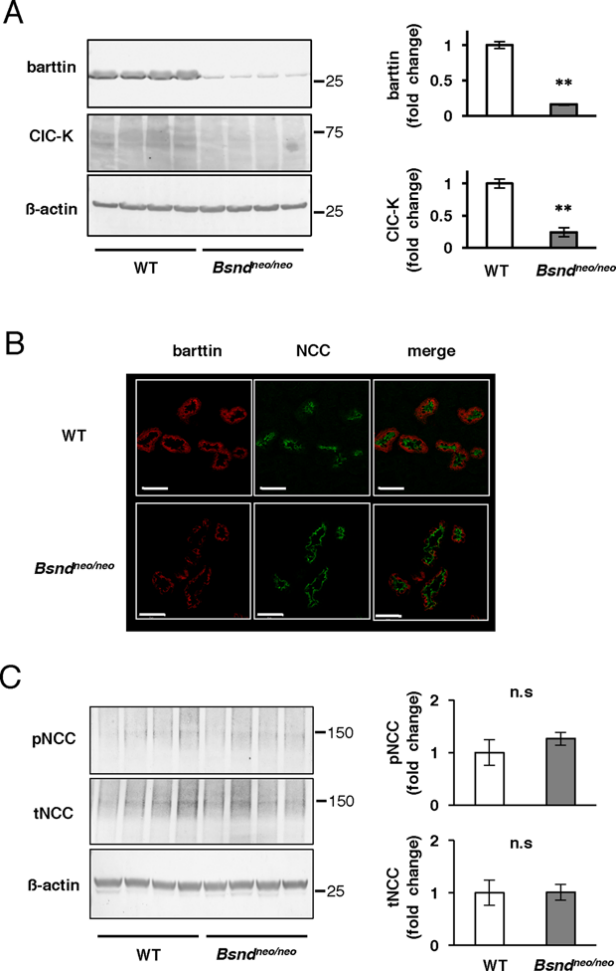
Baseline characteristics of *Bsnd^neo/neo^* mice (**A**) Representative immunoblots and densitometric analysis of barttin and ClC-K in the mice consuming a normal diet. The expression of barttin and ClC-K was quite low in the kidneys of *Bsnd^neo/neo^* mice; *n*=4. **P<0.005 by unpaired *t*test. (**B**) Representative immunofluorescences in the DCTs of WT mice and *Bsnd^neo/neo^* mice. In *Bsnd^neo/neo^* mice, barttin staining (red) was low and the localization upon the basolateral membrane was impaired. NCC (green) was stained as a marker of DCTs. Scale bars indicate 50 µm. (**C**) Representative immunoblots and densitometric analysis of NCC; *n*=4. Abbreviation: n.s., not significant.

Previously, we generated *Bsnd^neo/neo^* mice which contained a Neo-cassette and expressed a disease-causing R8L mutant of barttin [22]. Without deleting the Neo-cassette, the transcription of the *Bsnd* gene was significantly reduced. Unlike *Bsnd^−/−^* mice [25], *Bsnd^neo/neo^* mice could thrive to adulthood. In the present study, we confirmed that *Bsnd^neo/neo^* mice expressed only minimal amounts of barttin and ClC-K in their kidneys (Figure 1A). We performed immunofluorescence to investigate the amount and localization of barttin in DCT in *Bsnd^neo/neo^* mice. In *Bsnd^neo/neo^*mice, the staining of barttin was reduced in the DCT cells. Furthermore, basolateral staining of the R8L barttin mutant was clearly impaired (Figure 1B) as we previously reported [22]. These results suggested that ClC-K function in DCT cells was significantly reduced in *Bsnd^neo/neo^* mice.

We performed immunoblotting to determine whether *Bsnd^neo/neo^* mice on a normal diet have alterations in the NCC expression. No significant differences were observed in pNCC and tNCC between *Bsnd^neo/neo^* mice and WT mice (Figure 1C).

### The increase in NCC and SPAK phosphorylation in response to a low-K^+^ diet was significantly impaired in *Bsnd^neo/neo^* mice

To investigate the change of pNCC to K^+^ restriction, both WT mice and the *Bsnd^neo/neo^* mice were fed an HSNK diet or an HSLK diet same as a previous study [4]. Although general appearance of the mice consuming an HSLK diet were normal, *Bsnd^neo/neo^* mice started to lose weight after 3 days on an HSLK diet, which is probably due to potassium deficiency. Thus, we fed an HSLK diet or an HSNK diet, to the mice for only 2 days to avoid non-specific effects due to intolerance to HSLK diet. *Bsnd^neo/neo^* mice showed hypokalemia, metabolic alkalosis with low Cl^−^ and higher aldosterone levels when fed an HSNK diet (Table 1). In both WT mice and *Bsnd^neo/neo^* mice, plasma K^+^ levels were significantly reduced with an HSLK diet. There was no significant difference in the aldosterone level between *Bsnd^neo/neo^* and WT mice when compared with mice fed upon an HSLK diet.

**Table 1:** Blood data from mice receiving an HSNK diet or an HSLK diet for 2 days

	WT				Bsnd^neo/neo^			
	HSNK	*n*	HSLK	*n*	HSNK	*n*	HSLK	*n*
Na	148 ± 1	9	147 ± 2	7	147 ± 2	6	146 ± 3	7
K	4.7 ± 0.2	9	3.7 ± 0.2*	7	3.4 ± 0.1^†^	6	2.7 ± 0.2*^†^	7
Cl	118 ± 1	9	114 ± 1	7	108 ± 2^†^	6	107 ± 2^†^	7
tCO_2_	20 ± 1	6	20 ± 1	5	29 ± 1^†^	5	31 ± 2^†^	5
BUN	21 ± 2	6	17 ± 1	5	25 ± 2	5	25 ± 1^†^	5
Ht	42 ± 1	6	43 ± 2	5	48 ± 2	5	46 ± 2	5
Aldosterone	163 ± 39	5	98 ± 48	5	418 ± 81^†^	6	112 ± 66*	4

Values represent means ± S.E.M. Two-way ANOVA and Bonferroni’s test were performed to analyze statistical differences. *P<0.05, HSNK compared with HSLK in the same genotype. ^†^*P*<0.05, WT compared with*Bsnd^neo/neo^* mice on the same diet. Abbreviations: BUN, blood urea nitrogen (mg/dl); Cl, chloride (mmol/l); Ht, hematocrit (%), aldosterone (pg/ml); K, potassium (mmol/l); Na, sodium (mmol/l); tCO_2_, total CO_2_ (mmol/l).

**Figure 2:**
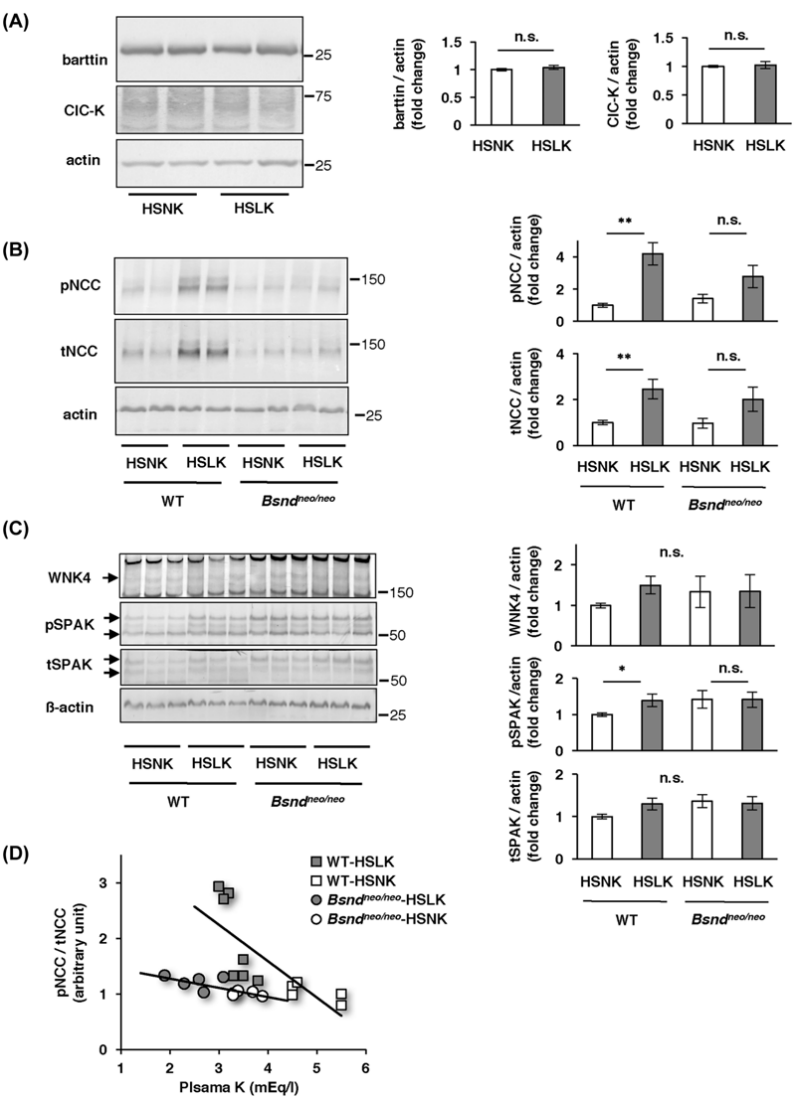
Relationship between plasma potassium and the WNK4-SPAK-NCC cascade (**A**) Representative immunoblots of barttin and ClC-K, and densitometric analysis in WT mice (*n*=6). (**B**) Representative immunoblots of NCC and densitometric analysis. WT-HSNK, *n*=8; WT-HSLK, *n*=9; *Bsnd^neo/neo^*-HSNK, *n*=7; *Bsnd^neo/neo^*-HSLK, *n*=7. (**C**) Representative immunoblots of WNK4 and SPAK, and densitometric analysis. Arrows on the left side of WNK4, pSPAK and tSPAK blots indicate WNK4, pSPAK and tSPAK bands (anti-pSPAK antibody from Dr Yang). Number of animals was same as (B). (**D**) Regression lines between the intensities of pNCC/tNCC and plasma K^+^ levels. Gray squares, open squares, gray circles, and open circles indicate data from WT-HSLK, WT-HSNK, *Bsnd^neo/neo^*-HSLK, and *Bsnd^neo/neo^*-HSNK groups, respectively. The slopes of the regression lines were significantly different (*P*<0.05). Response of pNCC and pSPAK to an HSLK diet was impaired in *Bsnd^neo/neo^* mice. *P<0.05 and **P<0.005 by Bonferroni’s test after two-way ANOVA. The linearity of protein detection for each antibody was confirmed in Supplementary Figures S2. Abbreviations: K, potassium; n.s., not significant.

We performed immunoblotting to evaluate the relative levels of WNK4-SPAK-NCC cascade. First, we analyzed the expression levels of barttin and ClC-K in the WT mice consuming an HSNK or HSLK diet, and we found that there was no significant difference in barttin and ClC-K between the diets (Figure 2A). Next, we found that both pNCC and tNCC were more abundant in the kidneys of WT mice consuming HSLK diet than those maintained upon HSNK diet (Figure 2B). In *Bsnd^neo/neo^* mice, the increase in pNCC and tNCC with HSLK diet was not enough to show a statistical difference (Figure 2B). The abundance of pSPAK and WNK4 in WT mice consuming HSLK diet showed significant increase and increasing tendency, respectively. However, the increase was not evident in *Bsnd^neo/neo^* mice (Figure 2C). The abundance of pNCC is known to correlate with plasma K^+^ concentration [6]. We found that pNCC/tNCC was well correlated with plasma K^+^ level in both WT mice (*r* = −0.76, *P*=0.0038) and *Bsnd^neo/neo^* mice (*r* = −0.68, *P*=0.0071) (Figure 2D). However, the slope of the regression lines was significantly greater in WT mice than in *Bsnd^neo/neo^* mice (*P*=0.020). These results indicate that the activation of WNK4-SPAK-NCC cascade in response to a low-K^+^ diet is dependent upon ClC-K function.

### In *ex vivo* kidney slice experiment, the increase in NCC phosphorylation in a low-K^+^ medium was not evident in *Bsnd^neo/neo^* mice

**Figure 3:**
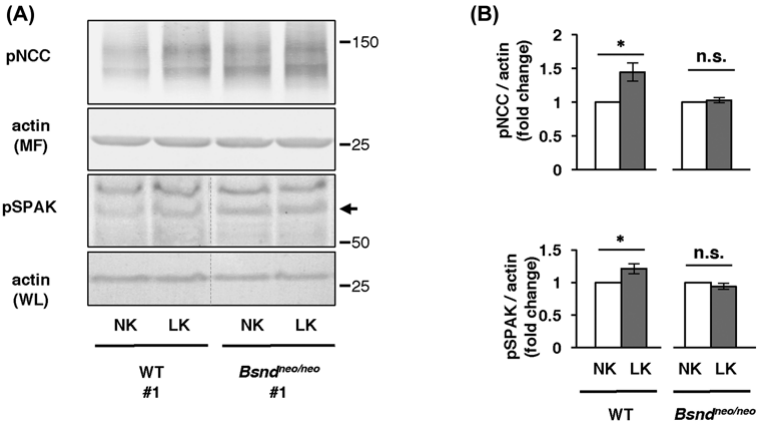
*Ex vivo* kidney slices experiment (**A**) Representative immunoblots of kidney slices incubated in a normal or low-K^+^ medium. (**B**) Densitometric analysis of pNCC and SPAK in kidney slices (new anti-pSPAK antibody). The slices from the same mouse kidney were incubated in a normal medium or a low-K^+^ medium. The fold change to a normal medium was analyzed by unpaired *t*test; *P<0.05, *n*=6. Open columns indicate normal medium groups, gray columns indicate low-K medium groups. The linearity of protein detection for each antibody was confirmed in Supplementary Figure S3. Abbreviations: LK, low potassium medium (K^+^ 2 mEq/l); NK, normal potassium medium (K^+^ 4 mEq/l); n.s., not significant.

To exclude non-specific and indirect effects on pNCC and pSPAK with HSLK diet in *Bsnd^neo/neo^* mice, we performed *ex vivo* kidney slice experiments, as previously performed [29]. In the kidney slices from WT mice, pNCC and pSPAK were significantly increased in a low-K^+^ medium, however, which was not observed in the kidney slices from *Bsnd^neo/neo^* mice (Figure 3). These data strongly supported our *in vivo* evidence that ClC-K is involved in SPAK and NCC activation in response to K^+^ restriction.

### *Bsnd^neo/ne^*^o^ mice did not show an elevation in blood pressure when fed an HSLK diet

**Figure 4:**
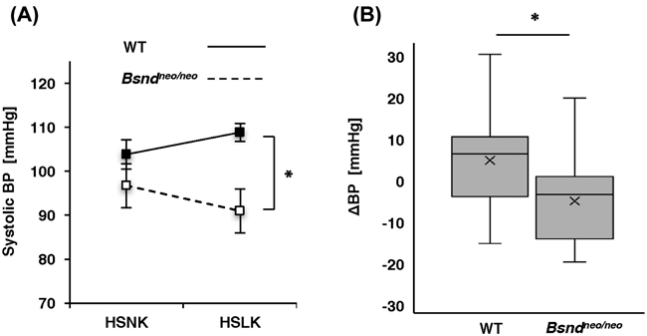
Systolic blood pressure measurement by tail cuffs At first, the mice were maintained on an HSNK diet, then the food was switched to an HSLK diet. BP were compared between the HSNK diet and the HSLK diet. (**A**) The average of systolic BP. BP from HSLK diet was significantly greater in WT than in *Bsnd^neo/neo^*. (**B**) A box-and-whisker plot of the change in BP between the diets. Boxes demonstrate median and 25–75% range; whiskers extend to the most extreme data point. The averages were shown as ‘x’s in the boxes. The change in BP was greater in WT than in *Bsnd^neo/neo^*. *P<0.05 according to the unpaired *t* test. *n*=14 (WT), *n*=11 (*Bsnd^neo/neo^*). Abbreviations: BP, blood pressure; HSLK, high-salt and low-potassium diet; ΔBP, change in BP.

To determine the contribution of ClC-K in the rise in blood pressure induced by a low-K^+^ diet, we compared the effects of diet to blood pressure between *Bsnd^neo/neo^* mice and WT mice. In WT mice, blood pressure showed increasing tendency (*n*=14, *P*=0.10), when fed an HSLK diet, but did not show an increase in *Bsnd^neo/neo^* mice (*n*=11, *P*=0.10) (Figure 4A). The change in blood pressure between the diets was significantly greater in the WT mice than in the *Bsnd^neo/neo^* mice (*P*=0.009) (Figure 4B). These data indicate that ClC-K plays a role in hypertension induced by a low-K^+^ diet, probably via the activation of WNK4, SPAK, and NCC.

## Discussion

Using *Bsnd^neo/neo^* mice, we demonstrated that ClC-K and barttin play an important role in the activation of WNK4-SAPK-NCC cascade in low-K^+^ conditions. When WT mice were fed an HSLK diet, the WNK4-SPAK-NCC cascade was activated, however, such activation did not occur in *Bsnd^neo/neo^* mice. In previous *in vitro* studies using HEK293 cells, loss-of-function mutant ClC-K2 transfection and a Cl^−^ channel inhibitor (DIDS) treatment showed a lower increase in pNCC in the response to a low-K^+^ condition [4]. Consistent with these findings *in vitro*, our *in vivo* findings strongly support the contribution of ClC-K2 in the mechanism underlying the low-K^+^-induced phosphorylation of NCC.

**Figure 5:**
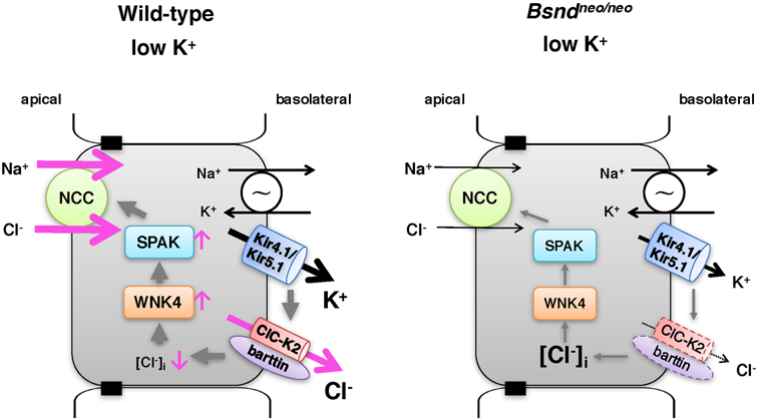
A scheme illustrating the mechanism Basolateral low extracellular potassium regulates the phosphorylation of the apical NCC in DCT cells of WT mice (left panel). In *Bsnd^neo/neo^* mice (right panel), NCC was not activated by low extracellular potassium.

The potential importance of the Cl^−^ channel in the regulation of WNK kinases is based on the idea that WNKs behave as Cl^−^-sensitive kinases [6,30]. WNKs have direct Cl^−^-binding sites in their catalytic sites, and these residues are conserved amongst WNKs [15,16]. The Cl^−^ ion binding to these sites inhibits autophosphorylation (= activity) of WNK kinases. It is thought that the negative basolateral membrane potential (hyperpolarization) is the main driving force for Cl^−^ to exit the cell [31]. In low-K^+^ condition, the Kir4.1/Kir5.1 complex makes the driving force because it is thought to be the predominant K^+^ channels in the basolateral membrane of DCT cells [32,33]. Indeed, the recent report about the doxycycline inducible kidney-specific *Kcnj10* knockout mouse showed that the lack of Kir4.1 (coded by *Kcnj10*) decreased the K^+^ reversal potential and basolateral Cl^−^ conductance in DCT cells [34]. In humans, loss-of-function mutations in the gene encoding Kir4.1 cause SeSAME/EAST syndrome, characterized by an electrolyte imbalance reminiscent of Gitelman syndrome, including salt wasting, hypocalciuria, hypomagnesemia, and hypokalemic metabolic alkalosis [35]. Because ClC-K2/b is the main Cl^−^ channel in the basolateral membrane of DCT cell [21], it is expected that the lack of ClC-K2/b disrupts the Cl^−^ exit in response to the change of [K^+^]_ex_. Indeed, although we could not analyze a change in [Cl^−^]_i_ in DCT cells, our finding that the *Bsnd^neo/neo^* mice did not show an increase in pSPAK after consuming a low-K^+^ diet strongly supports this hypothesis. We designed the scheme in Figure 5.

Furthermore, we found that a low-K^+^ diet induced an increase in blood pressure in WT mice, which was not observed in the *Bsnd^neo/neo^* mice. This indicates that ClC-K2/b contributes to the low-K^+^-induced hypertension via the WNK4-SPAK-NCC cascade. Because the elevation of blood pressure by the low-K^+^ diets was related to cardiovascular events and mortality [3], our findings suggested that CLC-K2/b could also be a drug target to decrease cardiovascular events as well as blood pressure.

A previous study clearly showed the decrease in NCC in ClC-K2 KO mice on the normal diet [21]. This result indicates the importance of ClC-K2 in NCC expression. However, the *Bsnd^neo/neo^* mice on the normal diet did not show a decrease in NCC (Figure 1C). One possible explanation for the discrepancy is that a minimal expression of ClC-K2 in *Bsnd^neo/neo^* mice might be enough for NCC expression. A previous experiment using isolated distal tubules showed that the Cl^−^ channel inhibitor NPPB had no effect on basolateral resting membrane potential [36]. Thus, it seems that the Cl^−^ efflux via ClC-K2 mainly occurs when a negative basolateral potential is generated by K^+^ channels, and the contribution of ClC-K2 for NCC expression on normal diet might be minimal.

In summary, *Bsnd^neo/neo^* mice consuming a low-K^+^ diet showed a blunted activation of the WNK4-SPAK-NCC cascade and a lesser increase in blood pressure. ClC-K2/b and barttin play important roles in low-K^+^-induced phosphorylation of NCC and regulation of blood pressure via the WNK4-SPAK cascade.

## Supporting information

**Figure S1:** **Confirmation of phospho-specific-SPAK antibody *in vivo***. Immunoblot of kidney homogenate from wild-type (left lane) and SPAK knockout mouse (right lane) with a phospho-specific SPAK antibody. The disappearance of bands from a SPAK knockout mouse confirms the specificity of our antibody (shown with arrows).

**Figure S2:** **Confirmation of our protein amount detection system by Western blotting in Figure 2.** The same loading amount of proteins as used in Figure 2 were set to 1. Half and quarter amount of protein was loaded together and the signal intensity was evaluated. (A) Representative immunoblots. (B) Correlation between the signal intensity and protein amount. Means with SEM were shown in the graphs. N = 4.

**Figure S3:** **Confirmation of our protein amount detection system by Western blotting in Figure 3.** The same loading amount of proteins as used in Figure 3 were set to 1. Half and quarter amount of protein was loaded together and the signal intensity was evaluated. (A) Representative immunoblots. (B) Correlation between the signal intensity and protein amount. Means with SEM were shown in the graphs. N = 4.

**Table S1:** List of antibodies

## Abbreviations

DCT: distal convoluted tubule
DIDS: 4,4′-diisothiocyanato-2,2′-stilbenedisulfonic acid disodium salt
HSLK: high-salt and low-K
HSNK: high-salt and normal-K
KO: knockout
NCC: sodium chloride cotransporter
NPPB: 5-Nitro-2-(3-phenylpropylamino)benzoic acid
OSR1: oxidative stress-related kinase 1
SPAK: STE20/SPS1-44 related proline/alanine-rich kinase
tNCC: total NCC
tSPAK: total SPAK
WNK: with-no-lysine kinase
WT: wilsd-type

## References

[1] Mente A., O’Donnell M.J., Rangarajan S., McQueen M.J., Poirier P., Wielgosz A. (2014) Association of urinary sodium and potassium excretion with blood pressure. N. Engl. J. Med. 371, 601–611 10.1056/NEJMoa1311989 25119606

[2] Riphagen I.J., Gijsbers L., van Gastel M.D.A., Kema I.P., Gansevoort R.T., Navis G. (2016) Effects of potassium supplementation on markers of osmoregulation and volume regulation. J. Hypertens. 34, 215–220 10.1097/HJH.0000000000000786 26599222

[3] O’Donnell M., Mente A., Rangarajan S., McQueen M.J., Wang X., Liu L. (2014) Urinary sodium and potassium excretion, mortality, and cardiovascular events. N. Engl. J. Med. 371, 612–623 10.1056/NEJMoa1311889 25119607

[4] Terker A.S., Zhang C., McCormick J.A, Lazelle R.A, Zhang C., Meermeier N.P. (2015) Potassium modulates electrolyte balance and blood pressure through effects on distal cell voltage and chloride. Cell Metab. 21, 39–50 10.1016/j.cmet.2014.12.006 25565204 PMC4332769

[5] Castañeda-Bueno M., Cervantes-Perez L.G., Rojas-Vega L., Arroyo-Garza I., Vázquez N., Moreno E. (2014) Modulation of NCC activity by low and high K(+) intake: insights into the signaling pathways involved. Am. J. Physiol. Renal Physiol. 306, F1507–F1519 10.1152/ajprenal.00255.2013 24761002 PMC4059971

[6] Terker A.S., Zhang C., Erspamer K.J., Gamba G., Yang C. and Ellison D.H. (2016) Unique chloride-sensing properties of WNK4 permit the distal nephron to modulate potassium homeostasis. Kidney Int. 89, 127–134 10.1038/ki.2015.289 26422504 PMC4814375

[7] Takahashi D., Mori T., Nomura N., Khan M.Z.H., Araki Y., Zeniya M. (2014) WNK4 is the major WNK positively regulating NCC in the mouse kidney. Biosci. Rep. 34, 10.1042/BSR20140047 24655003 PMC4212913

[8] Wade J.B., Liu J., Coleman R., Grimm P.R., Delpire E. and Welling P.A. (2015) SPAK-mediated NCC regulation in response to low-K+ diet. Am. J. Physiol. Renal Physiol. 308, F923–F931 10.1152/ajprenal.00388.2014 25651563 PMC4398835

[9] Ferdaus M.Z., Barber K.W., López-Cayuqueo K.I., Terker A.S., Argaiz E.R., Gassaway B.M. (2016) SPAK and OSR1 play essential roles in potassium homeostasis through actions on the distal convoluted tubule. J. Physiol. 594, 4945–4966 10.1113/JP272311 27068441 PMC5009767

[10] Vitzthum H., Seniuk A., Schulte L.H., Müller M.L., Hetz H. and Ehmke H. (2014) Functional coupling of renal K+ and Na+ handling causes high blood pressure in Na^+^ replete mice. J. Physiol. 592, 1139–1157 10.1113/jphysiol.2013.266924 24396058 PMC3948568

[11] Wilson F.H., Disse-Nicodeme S., Choate K.A., Ishikawa K., Nelson-Willams C., Desitter I. (2001) Human hypertension caused by mutations in WNK kinases. Science 293, 1107–1112 10.1126/science.1062844 11498583

[12] Simon D.B., Nelson-Williams C., Bia M.J., Ellison D., Karet F.E., Molina A.M. (1996) Gitelman's variant of Bartter's syndrome, inherited hypokalaemic alkalosis, is caused by mutations in the thiazide-sensitive Na-Cl cotransporter. Nat. Genet. 12, 24–30 10.1038/ng0196-24 8528245

[13] Moriguchi T., Urushiyama S., Hisamoto N., Iemura S.I., Uchida S., Natsume T. (2005) WNK1 regulates phosphorylation of cation-chloride-coupled cotransporters via the STE20-related kinases, SPAK and OSR1. J. Biol. Chem. 280, 42685–42693 10.1074/jbc.M510042200 16263722

[14] Vitari A.C., Deak M., Morrice N.A. and Alessi D.R. (2005) The WNK1 and WNK4 protein kinases that are mutated in Gordon’s hypertension syndrome phosphorylate and activate SPAK and OSR1 protein kinases. Biochem. J. 391, 17 10.1042/BJ20051180 16083423 PMC1237134

[15] Bazúa-Valenti S., Chávez-Canales M., Rojas-Vega L., González-Rodríguez X., Vázquez N., Rodríguez-Gama A. (2015) The effect of WNK4 on the Na+-Cl- cotransporter is modulated by intracellular chloride. J. Am. Soc. Nephrol. 26, 1781–1786 10.1681/ASN.2014050470 25542968 PMC4520168

[16] Piala A.T., Moon T.M., Akella R., He H., Cobb M.H. and Goldsmith E.J. (2014) Chloride sensing by WNK1 involves inhibition of autophosphorylation. Sci. Signal. 7, ra41 10.1126/scisignal.2005050 24803536 PMC4123527

[17] Kobayashi K., Uchida S., Mizutani S., Sasaki S. and Marumo F. (2001) Intrarenal and cellular localization of CLC-K2 protein in the mouse kidney. J. Am. Soc. Nephrol. 12, 1327–1334 11423561 10.1681/ASN.V1271327

[18] Yoshikawa M., Uchida S., Yamauchi A, Miyai A, Tanaka Y., Sasaki S. (1999) Localization of rat CLC-K2 chloride channel mRNA in the kidney. Am. J. Physiol. 276, F552–F558 10198414 10.1152/ajprenal.1999.276.4.F552

[19] Nozu K., Iijima K., Kanda K., Nakanishi K., Yoshikawa N., Satomura K. (2010) The pharmacological characteristics of molecular-based inherited salt-losing tubulopathies. J. Clin. Endocrinol. Metab. 95, E511–E518 10.1210/jc.2010-0392 20810575

[20] Fukuyama S., Okudaira S., Yamazato S., Yamazato M. and Ohta T. (2003) Analysis of renal tubular electrolyte transporter genes in seven patients with hypokalemic metabolic alkalosis. Kidney Int. 64, 808–816 10.1046/j.1523-1755.2003.00163.x 12911530

[21] Hennings J.C., Andrini O., Picard N., Paulais M., Huebner A.K., Cayuqueo I.K.L. (2017) The ClC-K2 chloride channel is critical for salt handling in the distal nephron. J. Am. Soc. Nephrol. 28, 209–217 10.1681/ASN.2016010085 27335120 PMC5198284

[22] Nomura N., Tajima M., Sugawara N., Morimoto T., Kondo Y., Ohno M. (2011) Generation and analyses of R8L barttin knockin mouse. Am. J. Physiol. Renal Physiol. 301, F297–F307 10.1152/ajprenal.00604.2010 21593186

[23] Birkenhäger R., Otto E., Schürmann M.J., Vollmer M., Ruf E.M., Maier-Lutz I. (2001) Mutation of BSND causes Bartter syndrome with sensorineural deafness and kidney failure. Nat. Genet. 29, 310–314 10.1038/ng752 11687798

[24] Estévez R., Boettger T., Stein V., Birkenhäger R., Otto E., Hildebrandt F. (2001) Barttin is a Cl- channel β-subunit crucial for renal Cl- reabsorption and inner ear K+ secretion. Nature 414, 558–561 10.1038/35107099 11734858

[25] Rickheit G., Maier H., Strenzke N., Andreescu C.E., De Zeeuw C.I., Muenscher A. (2008) Endocochlear potential depends on Cl- channels: mechanism underlying deafness in Bartter syndrome IV. EMBO J. 27, 2907–2917 10.1038/emboj.2008.203 18833191 PMC2580783

[26] Sohara E., Rai T., Yang S.-S., Ohta A., Naito S., Chiga M. (2011) Acute insulin stimulation induces phosphorylation of the Na-Cl cotransporter in cultured distal mpkDCT cells and mouse kidney. PLoS ONE 6, e24277 10.1371/journal.pone.0024277 21909387 PMC3164195

[27] Yang S., Morimoto T., Rai T., Chiga M., Sohara E., Ohno M. (2007) Molecular pathogenesis of pseudohypoaldosteronism type II: generation and analysis of a Wnk4(D561A/+) knockin mouse model. Cell Metab. 5, 331–344 10.1016/j.cmet.2007.03.009 17488636

[28] Nomura N., Nunes P., Bouley R., Nair A.V, Shaw S., Ueda E. (2014) High-throughput chemical screening identifies AG-490 as a stimulator of aquaporin 2 membrane expression and urine concentration. Am. J. Physiol. Cell Physiol. 307, C597–C605 10.1152/ajpcell.00154.2014 24944200 PMC4187055

[29] Penton D., Czogalla J., Wengi A., Himmerkus N., Loffing-Cueni D., Carrel M. (2016) Extracellular K + rapidly controls NCC phosphorylation in native DCT by Cl-dependent and -independent mechanisms. J. Physiol. 594, 6319–6331 27457700 10.1113/JP272504 PMC5088235

[30] Naito S., Ohta A., Sohara E., Ohta E., Rai T., Sasaki S. (2011) Regulation of WNK1 kinase by extracellular potassium. Clin. Exp. Nephrol. 15, 195–202 10.1007/s10157-010-0378-9 21107632

[31] Zaika O., Tomilin V., Mamenko M., Bhalla V. and Pochynyuk O. (2016) New perspective of ClC-Kb/2 Cl − channel physiology in the distal renal tubule. Am. J. Physiol. Renal Physiol. 310, F923–F930 10.1152/ajprenal.00577.2015 26792067 PMC5002062

[32] Zhang C., Wang L., Zhang J., Su X., Lin D., Scholl U.I. (2014) KCNJ10 determines the expression of the apical Na-Cl cotransporter (NCC) in the early distal convoluted tubule (DCT1). Proc. Natl. Acad. Sci. U.S.A. 111, 6–11 10.1073/pnas.1411705111 PMC4136599 25071208

[33] Lourdel S., Paulais M., Cluzeaud F., Bens M., Tanemoto M., Kurachi Y. (2002) An inward rectifier K(+) channel at the basolateral membrane of the mouse distal convoluted tubule: similarities with Kir4-Kir5.1 heteromeric channels. J. Physiol. 538, 391–404 10.1113/jphysiol.2001.012961 11790808 PMC2290070

[34] Cuevas C.A., Su X.-T., Wang M.-X., Terker A.S., Lin D.-H., McCormick J.A. (2017) Potassium sensing by renal distal tubules requires Kir4.1. J. Am. Soc. Nephrol. 28, 1814–1825 10.1681/ASN.2016090935 28052988 PMC5461801

[35] Bockenhauer D., Feather S., Stanescu H.C., Bandulik S., Zdebik A.A., Reichold M. (2009) Epilepsy, ataxia, sensorineural deafness, tubulopathy, and KCNJ10 mutations. N. Engl. J. Med. 360, 1960–1970 10.1056/NEJMoa0810276 19420365 PMC3398803

[36] Zaika O., Mamenko M., Boukelmoune N. and Pochynyuk O. (2015) IGF-1 and insulin exert opposite actions on ClC-K2 activity in the cortical collecting ducts. Am. J. Physiol. Renal Physiol 308, F39–F48 10.1152/ajprenal.00545.2014 25339702 PMC4281695

