# Supplementary material for: Role of ClC-K and barttin in low potassium-induced sodium chloride cotransporter activation and hypertension in mouse kidney
Source: Biosci Rep. 2018 Jan 30;38(1):BSR20171243. doi: 10.1042/BSR20171243 (PMC5789154; doi:10.1042/BSR20171243)
Supplement: Supplementary file 1 [file bsr20171243_Supp1.pdf]

Supplementary Information

Supplementary Material and Methods

Western blotting

Kidneys were lysed in detergent-free buffer {250 mM sucrose, 10 mM Triethanolamine, 1 mM EGTA, 1 mM EDTA, 1 mM Na orthovanadate, 50 mM Na fluoride, complete protease inhibitor cocktail (Roche)} with a homogenizer, and then the homogenates were centrifuged to separate to entire kidney samples without the nuclear fraction, as either whole kidney lysates (600 g, supernatant) and crude membrane fraction (17000 g, pellet). Samples were mixed with ß-ME Sample Treatment for Tris SDS (Cosmo Bio, Tokyo, Japan), and incubated for 20 min at 60 ^{o}C. Protein concentration was determined using Bradford ULTRA (Expedeon, UK). Protein samples were separated by SDS-PAGE on 5-20% gradient gels in Tris-Glysin SDS running buffer (Takara Bio Inc., Japna). Proteins were transferred to nitrocellulose membranes in transfer buffer (100 mM Tris, 192 mM Glysine). Membranes were blocked with 5% skim milk in TBST for 30 min at room temperature. Primary

antibodies (Table S1) were diluted in TBST. The membrane was incubated with a primary antibody overnight at 4 ^{o}C. Alkaline phosphatase-conjugated anti-IgG antibodies (anti-rabbit Cat. #S3738, Promega, anti-guinea pig Cat. #A2293, Sigma) as for secondary antibodies were diluted in 5% skim milk in TBST. After 3 times wash with TBST, the membrane was incubated with a secondary antibody for 45 min at room temperature. After 3 times wash with TBST, Western Blue (Promega) was used to detect the signals. The relative intensities of immunoblot bands were analyzed and quantified using ImageJ software (National Institutes of Health).

Table S1. List of antibodies

Protein	Host	Source [Reference] (Cat #/Lot#))	Loading protein (μg) *1	Dilution	Dilution medium
Barttin	Rabbit	[1]	2	1:1000 (WB) /1:500 (IF)	TBST(WB) /PBS(IF)
ClC-K	Guinea pig	Dr. Jentsch TJ (Gift) [2]	30	1:200	TBST
pNCC (Ser 71)	Rabbit	[3]	30 / 50	1:500	TBST
tNCC	Guinea pig	[1,4]	N/A	1:500 (IF)	PBS (IF)
tNCC	Rabbit	[4]	30	1:500 (WB)	TBST(WB)
WNK4	Rabbit	[4]	30	1:400	TBST
pSPAK (Ser 383)	Rabbit	Dr. Yang SS (Gift) [5]	30	1:500	Can get signal *2
pSPAK (Ser 383)	Rabbit	Newly generated	50 (ex vivo)	1:500	Can get signal *2
tSPAK	Rabbit	Cell signaling (#2281/Lot 2)	30	1:500	Can get signal *2
actin	Rabbit	Cytoskeleton (AAN01, Lot121)	30 / 50	1:1000	TBST
Rabbit IgG AP conjugate	N/A	Promega (S3738) /246053	N/A	1:7500	5% skim milk in TBST/Can get signal*2 (for SPAK)
Guinea pig IgG AP conjugate	N/A	Sigma (A2293) /10K4845	N/A	1:500	5% skim milk in TBST
Alexa-Rabbit IgG 488	Goat	Molecular Probes (A11008/57099A)	N/A	1:200	0.1% BSA in PBS
Alexa-Guinea pig IgG 546	Goat	Molecular Probes (A11007/1073002)	N/A	1:200	0.1% BSA in PBS

*1. in vivo / ex vivo experiment

*2. Can Get Signal Immunoreaction Enhancer Solution (TOYOBO, Tokyo, Japan)

WB: Western blotting, IF: immunofluorescence

References

1 Nomura, N., Tajima, M., Sugawara, N., Morimoto, T., Kondo, Y., Ohno, M., Uchida, K., Mutig, K., Bachmann, S., Soleimani, M., et al. (2011) Generation and analyses of R8L barttin knockin mouse. Am. J. Physiol. Renal Physiol. **301**, F297-307.

2 Vandewalle, A., Cluzeaud, F., Bens, M., Kieferle, S., Steinmeyer, K. and Jentsch, T. J. (1997) Localization and induction by dehydration of ClC-K chloride channels in the rat kidney. Am. J. Physiol. **272**, F678-88.

3 Yang, S., Morimoto, T., Rai, T., Chiga, M., Sohara, E., Ohno, M., Uchida, K., Lin, S., Moriguchi, T., Shibuya, H., et al. (2007) Molecular pathogenesis of pseudohypoaldosteronism type II: generation and analysis of a Wnk4(D561A/+) knockin mouse model. Cell Metab. **5**, 331–44.

4 Takahashi, D., Mori, T., Nomura, N., Khan, M. Z. H., Araki, Y., Zeniya, M., Sohara, E., Rai, T., Sasaki, S. and Uchida, S. (2014) WNK4 is the major WNK positively regulating NCC in the mouse kidney. Biosci. Rep. **34**, c.

5 Sohara, E., Rai, T., Yang, S.-S., Ohta, A., Naito, S., Chiga, M., Nomura, N., Lin, S.-H., Vandewalle, A., Ohta, E., et al. (2011) Acute insulin stimulation induces phosphorylation of the Na-Cl cotransporter in cultured distal mpkDCT cells and mouse kidney. PLoS One **6**, e24277.

Supplementary Figure legends

Figure S1. Confirmation of phospho-specific-SPAK antibody *in vivo*. Immunoblot of kidney homogenate from wild-type (left lane) and SPAK knockout mouse (right lane) with a phospho-specific SPAK antibody. The disappearance of bands from a SPAK knockout mouse confirms the specificity of our antibody (shown with arrows).

Figure S2. Confirmation of our protein amount detection system by Western blotting in Figure 2. The same loading amount of proteins as used in Figure 2 were set to 1. Half and quarter amount of protein was loaded together and the signal intensity was evaluated. (A) Representative immunoblots. (B) Correlation between the signal intensity and protein amount. Means with SEM were shown in the graphs. N = 4.

Figure S3. Confirmation of our protein amount detection system by Western blotting in Figure 3. The same loading amount of proteins as used in Figure 3 were set to 1. Half and quarter amount of protein was loaded together and the signal intensity was evaluated. (A) Representative immunoblots. (B) Correlation between the signal intensity and protein amount. Means with SEM were shown in the graphs. N = 4.

Figure S1

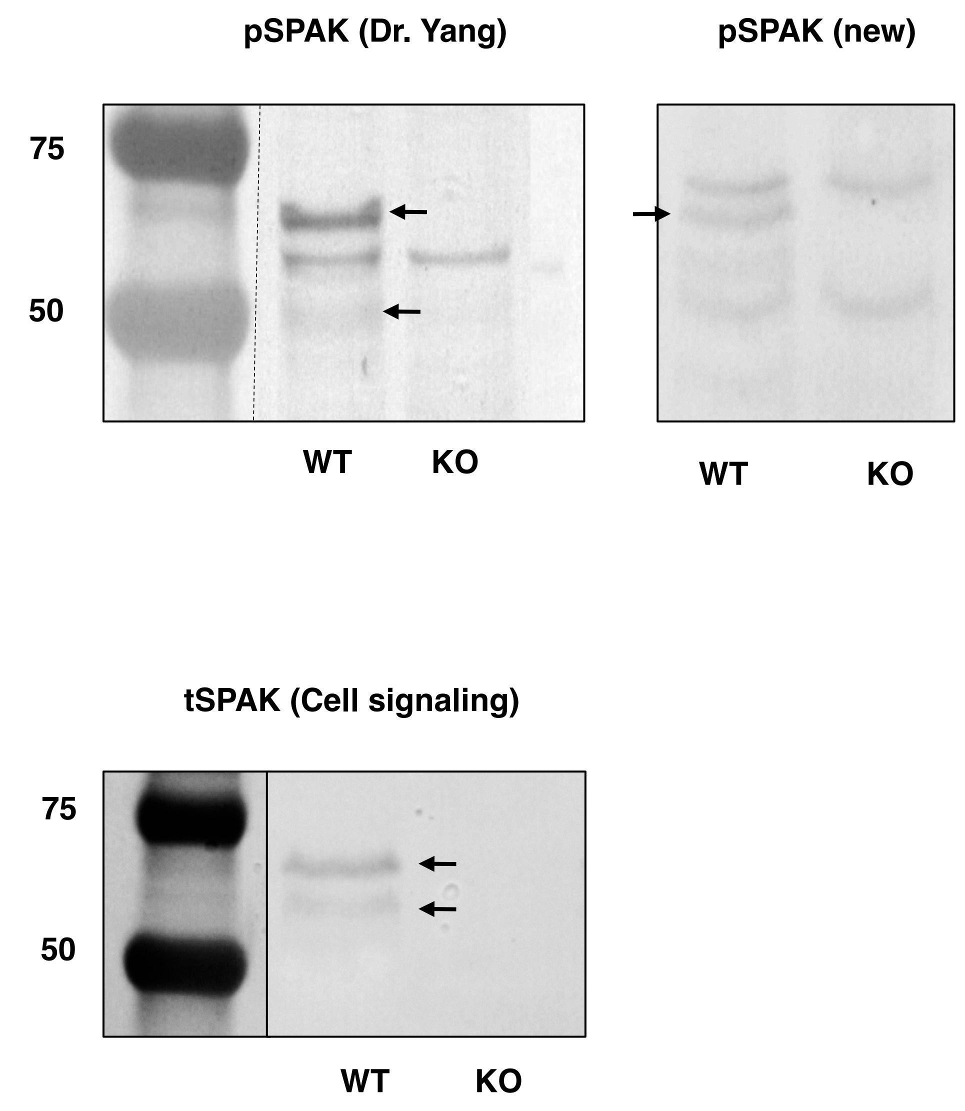

pSPAK (Dr. Yang)
pSPAK (new)
75
50
WT
KO
WT
KO
tSPAK (Cell signaling)
75
50
WT
KO

Figure S2

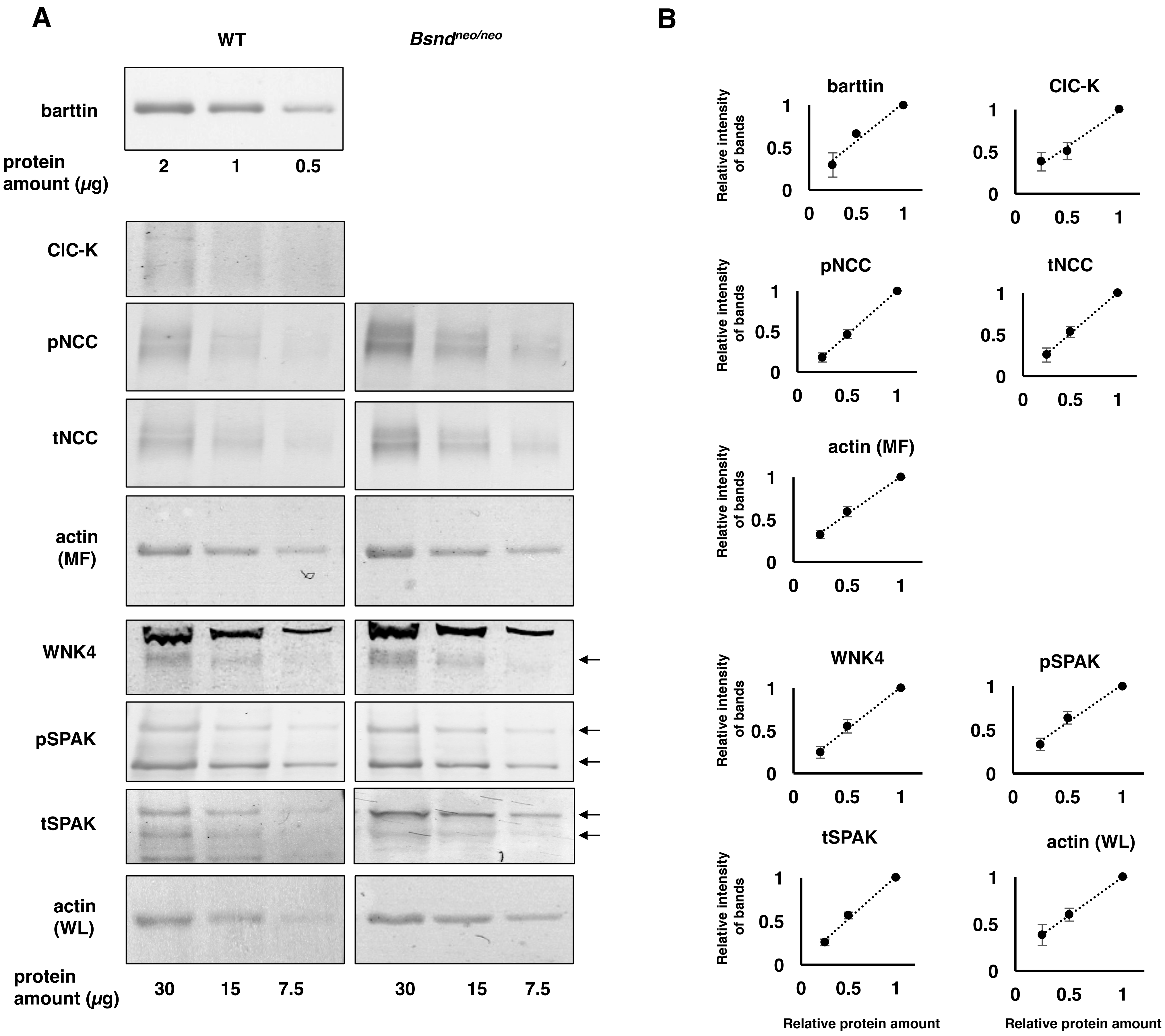
A
WT
Bsnd neo/neo
barttin
protein amount (μg)
2
1
0.5
ClC-K
pNCC
tNCC
actin (MF)
WNK4
pSPAK
tSPAK
actin (WL)
protein amount (μg)
30
15
7.5
30
15
7.5
B
barttin
ClC-K
pNCC
tNCC
actin (MF)
WNK4
pSPAK
tSPAK
actin (WL)
Relative intensity of bands
Relative protein amount
0
0.5
1

Figure S3

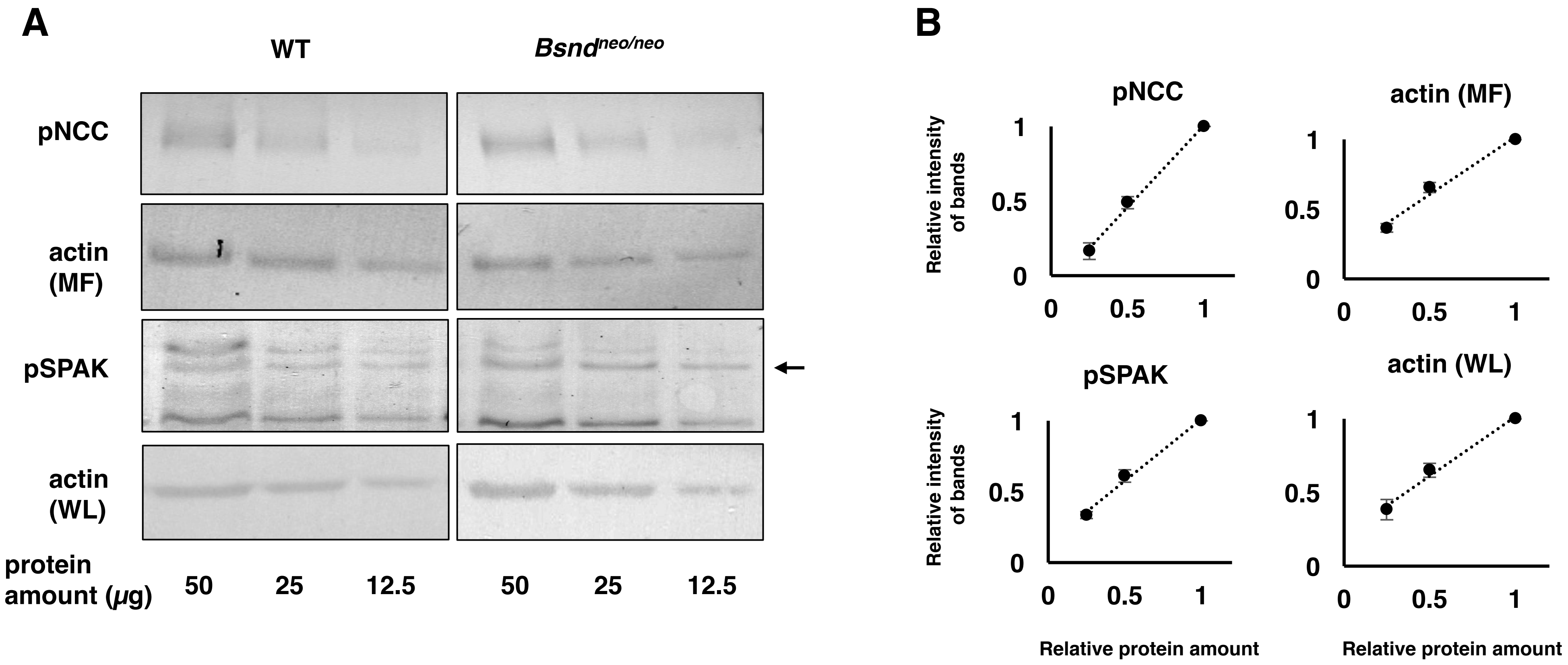

A
WT
Bsnd^neo/neo
pNCC
actin (MF)
pSPAK
actin (WL)
protein amount (μg)
50
25
12.5
50
25
12.5
B
pNCC
actin (MF)
pSPAK
actin (WL)
Relative intensity of bands
Relative protein amount
0
0.5
1
